# Osterix represses adipogenesis by negatively regulating PPARγ transcriptional activity

**DOI:** 10.1038/srep35655

**Published:** 2016-10-18

**Authors:** Younho Han, Chae Yul Kim, Heesun Cheong, Kwang Youl Lee

**Affiliations:** 1 College of Pharmacy & Research Institute of Drug Development, Chonnam National University, Gwangju, Republic of Korea; 2 Research Institute, National Cancer Center, 323 Ilsan-ro, Ilsandong-gu, Goyang-si, Gyeonggi-do 410-769, South Korea

## Abstract

Osterix is a novel bone-related transcription factor involved in osteoblast differentiation, and bone maturation. Because a reciprocal relationship exists between adipocyte and osteoblast differentiation of bone marrow derived mesenchymal stem cells, we hypothesized that Osterix might have a role in adipogenesis. Ablation of Osterix enhanced adipogenesis in 3T3-L1 cells, whereas overexpression suppressed this process and inhibited the expression of adipogenic markers including CCAAT/enhancer-binding protein alpha (C/EBPα) and peroxisome proliferator-activated receptor gamma (PPARγ). Further studies indicated that Osterix significantly decreased PPARγ-induced transcriptional activity. Using co-immunoprecipitation and GST-pull down analysis, we found that Osterix directly interacts with PPARγ. The ligand-binding domain (LBD) of PPARγ was responsible for this interaction, which was followed by repression of PPARγ-induced transcriptional activity, even in the presence of rosiglitazone. Taken together, we identified the Osterix has an important regulatory role on PPARγ activity, which contributed to the mechanism of adipogenesis.

Obesity, characterized by excessive fat deposition due to an energy imbalance between energy intake and expenditure, is a prevalent nutritional disorder, which tends to increase the risk of cardiovascular diseases, diabetes, and several types of cancer^1^,^2^. Obesity is a worldwide epidemic, with the prevalence of this condition steadily rising^3^. Therefore, there is a major need for therapeutic anti-obesity products that are proven to be safe and effective.

Obesity is also biologically defined as an excess adipose tissue mass, resulting from increased adipocyte numbers (hyperplasia) and increased adipocyte size (hypertrophy)^4^. Adipogenesis, the differentiation of mesenchymal stem cells into preadipocytes and the subsequent development into mature adipocytes, determines the number of adipocytes present in an organism^5^. Therefore, regulatory processes involved in adipogenesis are pivotal for obesity progression. To understand this process, the 3T3-L1 cell line has been established. In this model, confluent 3T3-L1 preadipocytes differentiate into adipocytes in response to hormonal stimulation and specific experimental conditions^6^. A cascade of sequential transcriptional factor activation is associated with regulatory events during adipogenesis. The earliest inductive event is driven by the concerted action of C/EBPβ and C/EBPδ in response to adipogenic stimuli in a combination with insulin, dexamethasone, and IBMX. These C/EBP transcription factors then stimulate expression of PPARγ and C/EBPα^7^. PPARγ and C/EBPα are major transcriptional activators that regulate each other to sustain their activity and induce adipocyte-specific gene activation to advance the formation of mature adipocytes^8^.

Osterix/Sp7, a zinc finger transcription factor, is commonly expressed in osteoblasts to enhance osteoblast differentiation and is required for proper osteoblast maturation during skeletal development^9^. Its expression is modulated by various factors, including insulin-like growth factor-I (IGF-I), bone morphogenetic proteins (BMPs), Msx2, and Runx2 during osteoblast differentiation^10^,^11^. Osterix interacts with the nuclear factor of activated T (NFAT) cells to form a complex, enhancing osteoblastic bone formation by activating the collagen type I α1 promoter^12^. Furthermore, Osterix deficient mice do not exhibit bone formation and are completely devoid of osteoblasts^9^. This suggests an indispensable function of Osterix in osteoblastogenesis.

Multipotent mesenchymal stem cells can differentiate into specific cell types including osteoblasts, adipocytes, skeletal myoblasts, chondrocytes, and fibroblasts that comprise connective tissue^13^. Differentiation into diverse cell lineages is regulated by several signaling pathways, various hormones, and different local factors^14^. Previous studies have revealed that several transcription factors regulate the determination of cell fate. In addition, accumulating evidence has demonstrated lineage switching, a large degree of cell plasticity, and a reciprocal relationship between cell lineages, during differentiation^15^,^16^,^17^. Down-regulation of Runx2, a master regulatory gene essential for osteoblasts, was shown to contribute to the acceleration of adipogenesis in 3T3-L1 cells^18^. In addition, nuclear factor I-C (NFI-C) was shown to stimulate osteoblast differentiation and new bone formation, while repressing adipogenesis via regulation of Osterix^19^, indicating that Osterix might have a negative role in adipogenesis. In the present research, we attempted to elucidate the functional roles of Osterix during adipogenesis by measuring the expression level of Osterix and the effect of Osterix overexpression and knockdown in 3T3-L1 cells. Finally, the regulatory mechanism of Osterix to PPARγ-mediated transcriptional activity was confirmed.

## Results

### Osterix is upregulated during the later stages of adipogenesis

**Figure 1:**
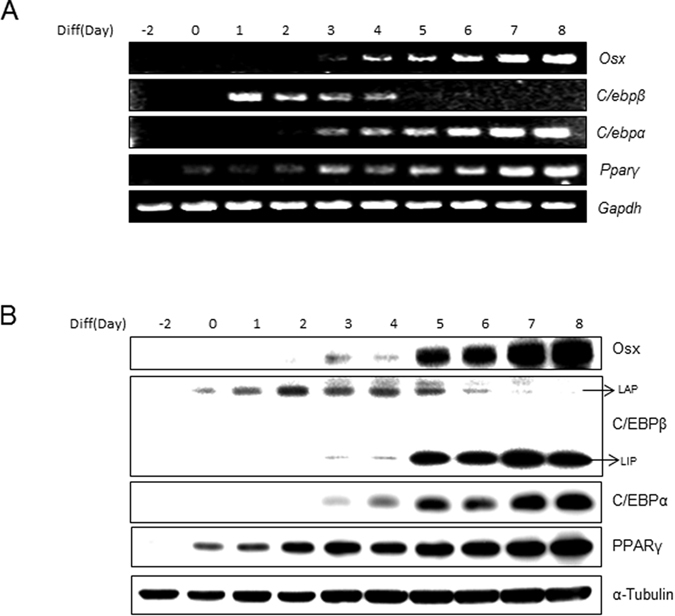
Expression of Osterix during adipogenesis in 3T3-L1 cells. (**A**,**B**) Adipogenesis of 3T3-L1 cells was induced with MDI. The mRNA and protein levels of Osterix (Osx), C/EBPβ, C/EBPα, and PPARγ at were compared at different time-points by RT-PCR and immunoblotting. *Gapdh* and α-tubulin were used as loading controls. Gene expression was quantified after 25 (*Gapdh)*, 30 (*Osterix*, *C/ebpα*, *and Pparγ*), and 35 cycles (*C/ebpβ*) of PCR. For C/EBPβ, liver-activating protein (LAP) and liver-inhibitory protein (LIP) are indicated.

To investigate the potential role of Osterix in adipogenesis, 3T3-L1 cells were used. This cell line completely reproduces the process of adipogenesis, with accompanying morphological changes and expression of adipogenic genes, and is thus a well-used and well-characterized *in vitro* model system. The pattern of Osterix abundance was examined by RT-PCR and western blotting in 3T3-L1 preadipocyte cells grown in adipogenic medium for 8 days. Our results indicated that *Osterix* mRNA was significantly increased from day 5 after treatment with adipogenic stimuli, reaching a maximum at the end of differentiation (Fig. 1A). As previously reported, the mRNA level of *Pparγ* and *C/ebpα*, which are transcription factors in the later stage of adipogenesis, was increased during the process of 3T3-L1 cell differentiation. In addition, the level of Osterix protein also steadily increased in a similar manner (Fig. 1B). These data demonstrate that the abundance of Osterix is increased during the later stages of adipogenic differentiation in 3T3-L1 cells.

### Overexpression of Osterix represses 3T3-L1 adipogenesis

**Figure 2:**
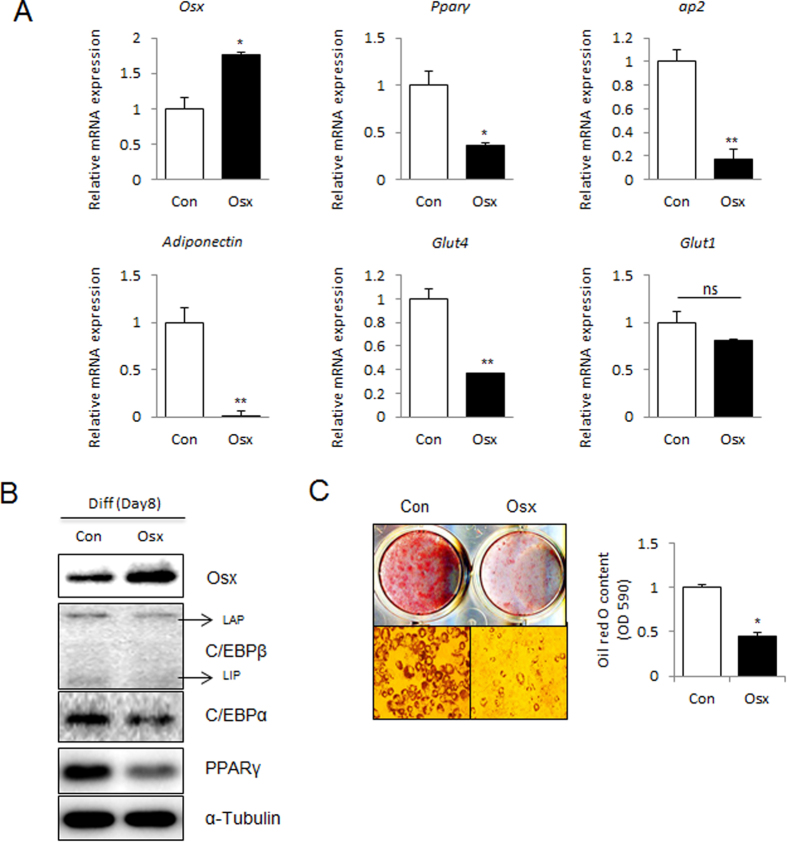
Overexpression of Osterix inhibits adipogenesis in 3T3-L1 cells. 3T3-L1 cells were transfected with empty vector (Con) or Myc-Osterix expressing plasmid (Osx). Cells were then cultured in adipogenic medium for 8 days. (**A**) The mRNA expression of *Osterix*, *Pparγ*, *ap2*, *Adiponectin*, *Glut4*, and *Glut1* was determined by real-time PCR and normalized to *Gapdh*. (**B**) The protein expression of Osterix, C/EBPβ, C/EBPα, and PPARγ was confirmed by immunoblotting. α-tubulin was used as a loading control. For C/EBPβ, liver-activating protein (LAP) and liver-inhibitory protein (LIP) are indicated. (**C**) Oil red O staining of lipid droplets in 3T3-L1 cells was performed at day 8 after adipogenic induction. The lipid accumulation was quantified by measuring the OD_530 nm_. *P < 0.05 and **P < 0.01 when compared to the control (Con); Student’s *t*-test, *n* = 3.

To determine the effect of Osterix on adipogenesis, 3T3-L1 cells were transiently transfected with Myc-tagged Osterix. Cells were then induced to differentiate into adipocytes with adipogenic medium for 8 days. At the end of differentiation, expression levels of various adipogenic markers were confirmed using real-time PCR and western blotting. Among the genes examined, the mRNA levels of *Pparγ*, *ap2*, *Adiponectin*, and *Glut4* were significantly attenuated in Osterix-overexpressing cells compared to that of control cells, whereas the abundance of *Glut1* was relatively unaffected by Osterix overexpression (Fig. 2A). Western blotting results showed that Osterix overexpression downregulated the abundance of C/EBPα and PPARγ proteins, which are the key transcription factor in adipogenesis (Fig. 2B). Moreover, oil red O staining on day 8 indicated that Osterix overexpression considerably suppressed the accumulation of lipid droplets (Fig. 2C). These results demonstrate that Osterix has an inhibitory role during 3T3-L1 adipogenesis.

### Knockdown of Osterix promotes 3T3-L1 adipogenesis

**Figure 3:**
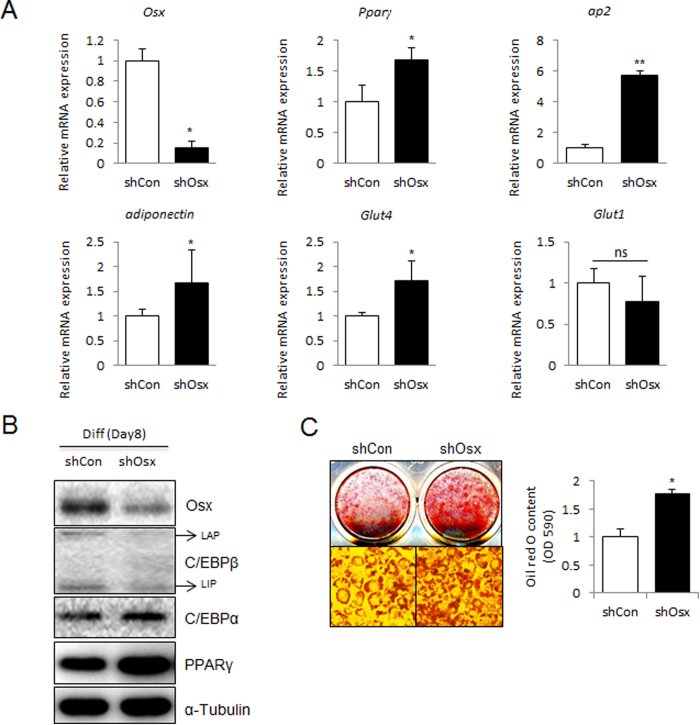
Knockdown of Osterix enhances adipogenesis in 3T3-L1 cells. 3T3-L1 cells were transfected with an empty pSuper vector (shCon), or pSuper-Osterix (shOsx). Cells were then cultured in adipogenic medium for 8 days. (**A**) The mRNA expression of *Osterix*, *Pparγ*, *ap2*, *Adiponectin*, *Glut4*, and *Glut1* was determined by real-time PCR and normalized to *Gapdh*. (**B**) The protein expression of Osterix, C/EBPβ, C/EBPα, and PPARγ was confirmed by immunoblotting. α-tubulin was used as a loading control. For C/EBPβ, liver-activating protein (LAP) and liver-inhibitory protein (LIP) are indicated. (**C**) Oil red O staining of lipid droplets in 3T3-L1 cells was performed at day 8 after adipogenic induction. The lipid accumulation was quantified by measuring the OD_530 nm_. *P < 0.05 and **P < 0.01 when compared to the control (shCon); Student’s *t*-test, *n* = 3.

To directly define the regulatory role of endogenous Osterix during adipogenesis, Osterix abundance was suppressed by shRNA, which was confirmed by quantitative real-time PCR and western blotting. Knockdown of Osterix promoted mRNA and protein abundance of some adipocyte-specific genes (Fig. 3A,B). Consistent with Osterix overexpression results, the level of *Glut1* was relatively unaffected by knockdown of Osterix. Moreover, lipid accumulation examined by oil red O staining was enhanced by Osterix knockdown (Fig. 3C). These data suggest that Osterix knockdown advances the adipogenic differentiation of 3T3-L1 cells.

### Osterix inhibits PPARγ-induced transcriptional activity

**Figure 4:**
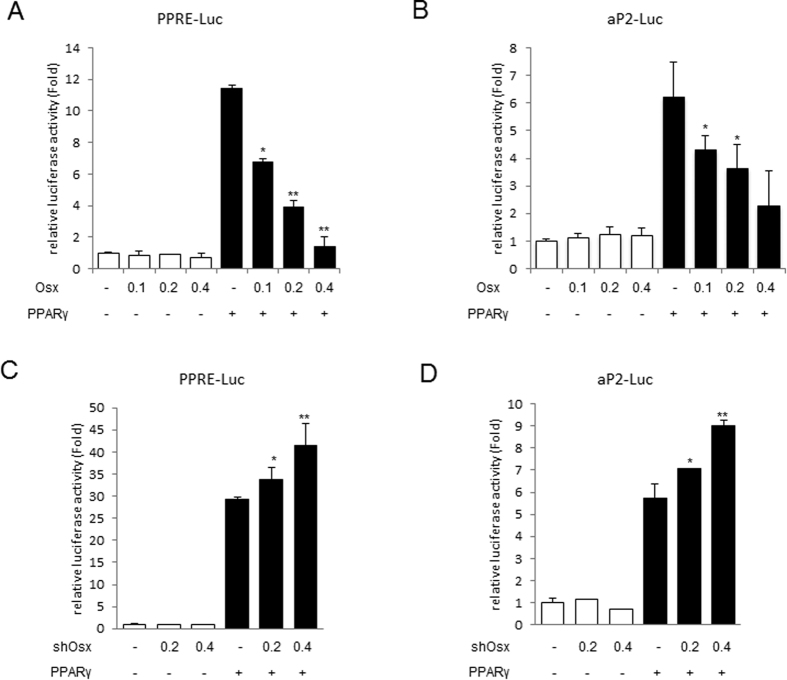
Osterix represses PPARγ-induced transcriptional activity. (**A**,**B**) 3T3-L1 cells were co-transfected with indicated combinations of HA-PPARγ (0.2 μg) and Myc-Osterix (0.1, 0.2, or 0.4 μg) along with a PPRE-Luc or aP2-Luc reporter. The expression of the reporter was measured using a luciferase assay. (**C**,**D**) 3T3-L1 cells were co-transfected with indicated combinations of HA-PPARγ (0.2 μg) and shRNA to Osterix (0.2 or 0.4 μg) along with the PPRE-Luc or aP2-Luc reporter. The expression of the reporter was measured using a luciferase assay. *P < 0.05 and **P < 0.01 compared to samples transfected with only PPARγ; ANOVA–Bonferroni *post hoc* test, *n* = 3.

Because Osterix was determined to have an important role in PPARγ expression, we proposed that PPARγ, a master transcription factor, is a potential target of Osterix during adipogenesis. To determine if Osterix could regulate PPARγ-induced transcriptional activity, 3T3-L1 cells were co-transfected with Osterix and PPARγ along with aP2-Luc or PPRE-Luc reporter. Both aP2-Luc and PPRE-Luc luciferase reporters contain PPARγ-responsive elements. As expected, PPARγ alone significantly induced the expression of aP2-Luc or PPRE-Luc reporter. When Osterix and PPARγ were co-expressed, Osterix significantly repressed PPARγ-induced transcriptional activity in a dose-dependent manner (Fig. 4A,B). To further investigate the inhibitory effect of endogenous Osterix, we investigated the effect of Osterix knockdown on the activity of the luciferase reporter. Compared to control, knockdown of Osterix enhanced PPARγ transcriptional activity in a dose-dependent manner for both the aP2-Luc and PPRE-Luc reporters (Fig. 4C,D). Taken together, these results indicate that Osterix inhibits the transcriptional activity of PPARγ.

### The ligand-binding domain of PPARγ is involved in its interaction with Osterix

**Figure 5:**
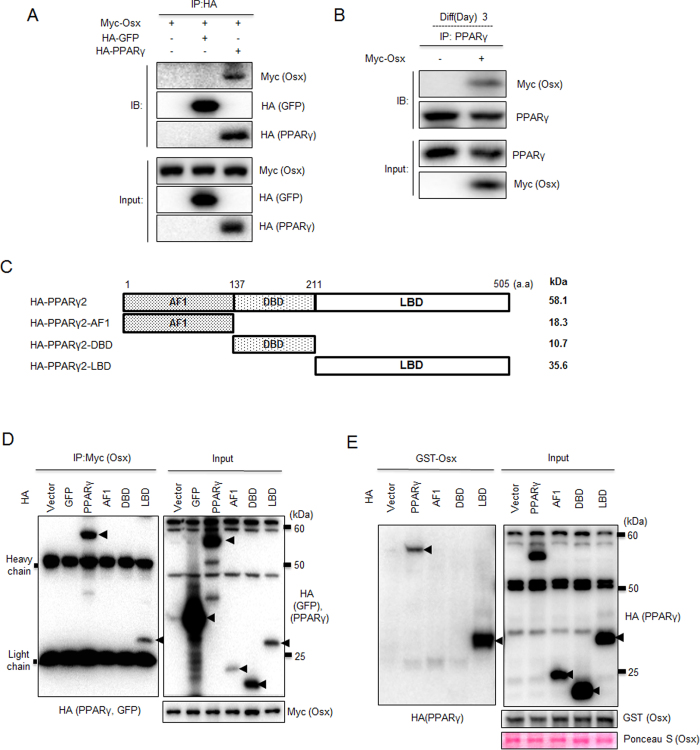
The LBD of PPARγ is involved in the interaction with Osterix. (**A**) 3T3-L1 cells were transfected with indicated expression plasmids. To examine the binding of Osterix and PPARγ, whole cell extracts were prepared and co-immnunoprecipitation was performed using HA antibody with subsequent western blotting using Myc or HA antibodies. (**B**) 3T3-L1 cells were transfected with Myc-Osterix plasmid or empty Myc vector. Cells were then cultured in MDI-containing differentiation medium for 3 days. The interaction between endogenous PPARγ and overexpressed Osterix is examined by IP using a PPARγ antibody followed by IB using a Myc antibody. (**C**) To identify the PPARγ domain that interacts with Osterix, a deletion construct of PPARγ was constructed. A depiction of the construct is shown here. (**D**) 3T3-L1 cells were transfected with indicated expression plasmids. To examine the binding of Osterix to the AF1, DBD, and LBD of PPARγ, co-immunoprecipitation assays were performed using a Myc antibody and western blotting was performed with Myc or HA antibodies. (**E**) Identification of the PPARγ domain that interacts with Osterix *in vitro*. The purified HA-tagged PPARγ deletion constructs were incubated with GST-Osx in separate incubation reactions and the bound proteins were identified by western blot analysis. Additionally, expression of GST proteins was confirmed by Ponceau S staining.

The above results imply the possibility of physical interaction between Osterix and PPARγ. Therefore, we further analyzed if the inhibitory effect of Osterix on PPARγ-induced transcriptional activity was due to physical interaction between these two proteins using co-immunoprecipitation (co-IP). Epitope-tagged Osterix interacted with overexpressed PPARγ in 3T3-L1 cells, but overexpressed GFP did not interact (Fig. 5A). Endogenous PPARγ, in MDI-stimulated 3T3-L1 cells, also interacted with overexpressed Osterix (Fig. 5B). To determine the domains of PPARγ responsible for the interaction with Osterix, we performed co-IP using Osterix and various PPARγ deletion mutants (Fig. 5C). When overexpressed in 3T3-L1 cells, the ligand-binding domain (PPARγ-LBD) of PPARγ interacted with Osterix, whereas the activation function 1 domain (PPARγ-AF1) and DNA binding domain (PPARγ-DBD) did not interact (Fig. 5D). We also examined the interaction between purified Osterix and PPARγ deletion mutants overexpressed in 3T3-L1 cells. Purified GST-tagged Osterix pulled down full-length PPARγ and PPARγ-LBD, but not PPARγ-AF1 or PPARγ-DBD (Fig. 5E). These results suggest the possibility of a direct interaction between Osterix and the PPARγ LBD.

### Osterix represses rosiglitazone-induced adipogenesis

**Figure 6:**
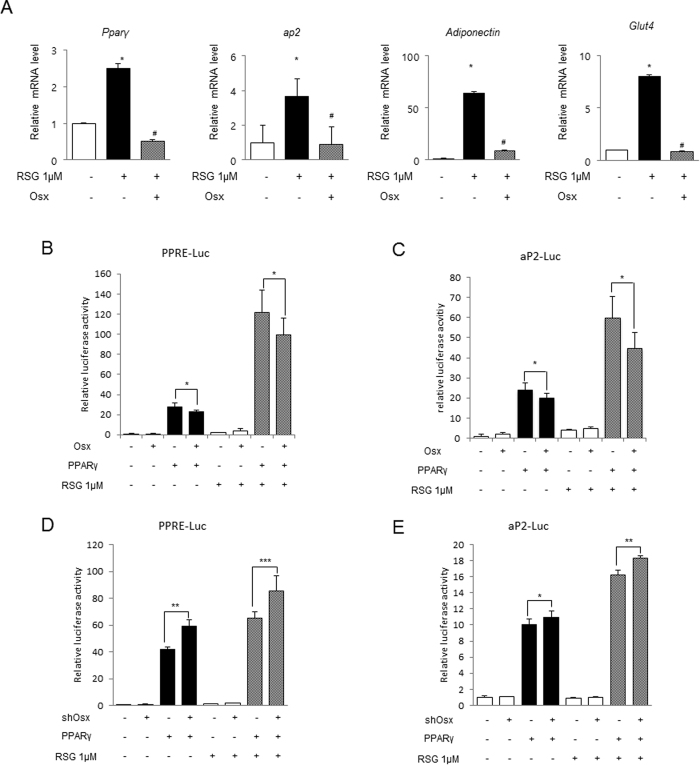
Osterix represses rosiglitazone-induced adipogenesis. (**A**) 3T3-L1 cells were transfected with an empty vector or Myc-Osterix expression plasmid. Cells were then cultured with or without 1 μM rosiglitazone (RSG) for 8 days. The mRNA expression of *Pparγ*, *ap2*, *Adiponectin*, and *Glut4* was determined by real-time PCR and normalized to that of *Gapdh*. *P < 0.05 compared to the control. ^#^*P* < 0.05 compared to samples treated only with rosiglitazone (RSG); ANOVA–Bonferroni *post hoc* test, *n* = 3. (**B**,**C**) 3T3-L1 cells were co-transfected with indicated combinations of HA-PPARγ (0.2 μg) and Myc-Osterix (0.2 μg) along with PPRE-Luc or aP2-Luc reporters. (**D**,**E**) 3T3-L1 cells were co-transfected with indicated combinations of HA-PPARγ (0.2 μg) and shRNA to Osterix (0.2 μg) along with PPRE-Luc or aP2-Luc reporter. After transfection (24 h), cells were treated with or without 1 μM rosiglitazone (RSG) and cultured for additional 12 h. The expression of the reporter was measured using a luciferase assay. *P < 0.05, **P < 0.01, and ***P < 0.001; Student’s *t*-test, *n* = 3.

As the above results indicated that the LBD of PPARγ interacted with Osterix, we hypothesized that Osterix could alter the ligand binding affinity of PPARγ. To examine this, we performed the following experiments using rosiglitazone, well known PPARγ agonist that stimulates adipogenesis via the activation with PPARγ^20^. As shown in Fig. 6A, rosiglitazone significantly enhanced mRNA production of various adipogenic markers including *Pparγ*, *ap2*, *Adiponectin*, and *Glut4*, and the expression of these factors was reduced by Osterix overexpression. In addition, the results of luciferase reporter assays showed that PPARγ-induced transcriptional activity was further enhanced upon stimulation by rosiglitazone, and Osterix effectively suppressed PPARγ transcriptional activities even in the presence of rosiglitazone (Fig. 6B,C). Furthermore, Osterix-knockdown increased PPARγ-induced, rosiglitazone-stimulated reporter expression (Fig. 6D,E). Clearly, these results imply that Osterix has the ability to suppress rosiglitazone-induced adipogenesis.

## Discussion

**Figure 7:**
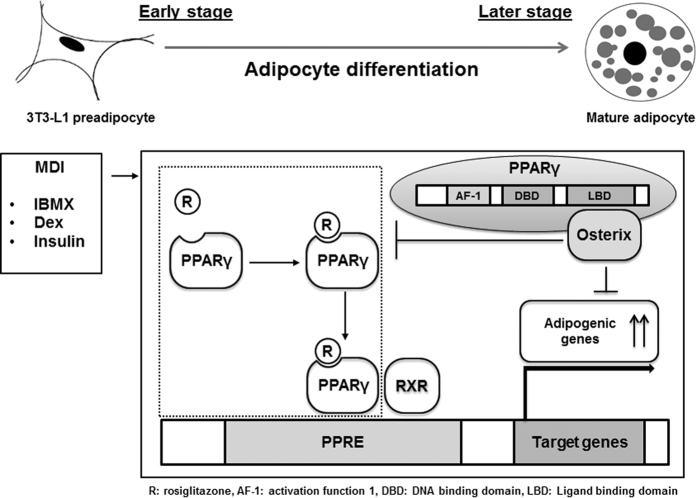
Working model for the regulatory effect Osterix in adipogenesis. Osterix down-regulated expression and transcriptional activity of PPARγ in the presence of rosiglitazone by direct interaction with ligand-binding domain (LBD) of PPARγ, and ultimately repressed adipogenesis.

In this study, we demonstrated the following. First, Osterix exerts a negative regulatory effect on adipogenesis of 3T3-L1 preadipocytes. Second, abundance of Osterix is increased at the later stage of adipogenesis, and Osterix knockdown enhances adipogenesis in 3T3-L1 cells. Third, Osterix represses PPARγ-induced transcriptional activity through direct physical interactions between these proteins. Fourth, the LBD of PPARγ is required for the interaction with Osterix, and finally, the ligand binding affinity of PPARγ is regulated by the action of Osterix (Fig. 7). These results demonstrate that Osterix is a negative modulator of transcriptional regulation, important for adipogenesis.

Adipogenesis requires both the down-regulation of adipogenic inhibitors and the induction of the pre-adipogenic factors, and is regulated by a cascade of adipogenic transcription factors, which include CCAAT-enhancer binding protein (C/EBP-α, -β and -δ) and peroxisome proliferator-activated receptor gamma (PPARγ)^21^. Upon stimulation with adipogenic conditions, preadipocyte cells begin the adipogenic differentiation cascade with the expression of C/EBPβ within several hours, followed by an induction of PPARγ and C/EBPα expression^22^. PPARγ and C/EBPα cooperatively activate the expression of adipogenic genes to further promote the formation of a mature adipocyte^23^. In our data, the expression of these factors was decreased upon Osterix overexpression (Fig. 2B). In addition, our findings indicate that Osterix can negatively regulate the transcriptional activity of PPARγ (Fig. 4). This may explain the decreased adipogenic differentiation in 3T3-L1 cells with overexpression of Osterix (Fig. 2C). Various intracellular signaling molecules act as anti-adipogenic factors, including Wnt10b, GATA2, 3, CHOP, and Pref-1^24^,^25^,^26^,^27^. The levels of all of these molecules are high in preadipocyte cells but are downregulated upon adipogenic stimuli at an early stage of differentiation. Interestingly, unlike previously reported anti-adipogenic factors, Osterix was not detected in preadipocytes, but was expressed at the later stage of differentiation (Fig. 1), suggesting that Osterix negatively regulates adipogenesis by repressing the PPARγ activity and inhibiting additional progression of differentiation.

In previous studies, it was shown that Osterix directly interacts with various proteins such as NO66 and RNA helicase A to modulate the functional activity of each protein^28^,^29^. In our data, the negative regulation of PPARγ activity by Osterix was supported by the physical interaction between these two proteins (Fig. 5A,B). PPARγ contains modular functional domains including an N-terminal transactivation domain (AF1), which is ligand-independent, a highly conserved DNA-binding domain (DBD), and a C-terminal ligand-binding domain (LBD). The LBD is responsible for the ligand-dependent transactivation function (AF2) that regulates ligand-dependent activation via interaction with receptor co-activators^30^,^31^. Among these domains, only the LBD of PPARγ was involved in the interaction with Osterix (Fig. 5D,E), indicating that Osterix could affect the ligand binding affinity of PPARγ. Indeed, Osterix overexpression suppressed rosiglitazone-induced expression of several adipogenic genes (Fig. 6A). In addition, further upregulation of PPARγ transcriptional activity in the presence of rosiglitazone was regulated by Osterix (Fig. 6B–E). These results suggest that Osterix could affect the ligand binding affinity PPARγ.

Mesenchymal stem cells have the potential to differentiate into osteoblasts, adipocytes, myocytes, and chondrocytes^32^, and accumulating evidence from other studies suggests that there are is a close relationship between osteoblasts and adipocytes during development^33^. Previous studies have suggested that master transcription factors, Runx2 and PPARγ, in osteoblasts and adipocytes respectively, are involved in the regulation of reciprocal differentiation processes. PPARγ haploinsufficiency in bone marrow progenitor cells enhances bone mass by stimulating osteoblast differentiation^34^. In contrast, Runx2-deficient calvarial cells fail to acquire osteoblastic phenotypes, but show enriched adipocyte differentiation potential^35^. Meanwhile, as a second transcription factor that is essential for osteoblast differentiation, repression of Osterix by dexamethasone committed to adipogenic differentiation in rat calvaria-derived cells^36^. In addition, it has been elucidated that a primate-specific microRNA targeting Osterix maintains adipocytes and osteoblast in the differentiation of human mesenchymal stem cells^37^. Integrating previous investigation with present our study, it can be proposed that Osterix has an important role a negative regulator of adipocyte differentiation.

In conclusion, the present study provides that Osterix directs an anti-adipogenic effect through the down-regulation of PPARγ expression and transcriptional activity. Given the above, it will be significant to elucidate the mechanisms controlling Osterix expression, which could lead to further identification of the mechanism that regulates adipogenesis.

## Materials and Methods

### Cell culture and Adipogenic differentiation in 3T3-L1 preadipocytes

Mouse preadipocyte 3T3-L1 cell line was grown in Dulbecco’s modified Eagle medium (DMEM, Life Technologies, Carlsbad, CA, USA) containing 10% bovine serum (BS, Gibco Invitrogen, Carlsbad, CA, USA) and 1% antibiotic-antimycotic (Thermo Fisher). Two-day postconfluent (day 0) cells were induced to differentiate with mixture of 0.5 mM 3-isobutyl-1-methylxanthine (Sigma, St. Louis, MO, USA), 1 μM dexamethasone (Sigma), and 10 μg/mL insulin (Sigma) in DMEM supplemented with 10% fetal bovine serum (FBS, Gibco Invitrogen) for two days. At this time, the medium consisting of DMEM with 10% FBS subsequently replaced every two days. Cells were observed for the accumulation of lipid droplets until day 8.

### Plasmids

For plasmids expressing Myc-Osterix, full length Osterix (mouse, 1287 bp: NCBI Reference Sequence: NP_569725.1) was subcloned with a Myc tag into CMV promoter-derived mammalian expression vector (pCS4). For plasmids expressing HA-PPARγ, full length PPARγ was subcloned with a HA tag into pCS4. Deletion of PPARγ was constructed by PCR-based mutagenesis method and confirmed by DNA sequence analysis. The HA-GFP plasmid was generously provided by Dr. Chang-Yeol Yeo (Ewha University, Seoul, Korea). The constructs for aP2-Luc^38^ and PPRE-Luc^39^ are described previously. For Osterix knockdown experiments, small hairpin RNA (shRNA) oligonucleotides were constructed by targeting a 19-base pair sequence (GT CTA CAC TTC CCT GGA TA) of the mouse Osterix gene. Annealed oligonucleotides were subcloned into the pSUPER.retro.puro vector (Oligoengine).

### Materials

Anti-C/EBPβ (04-1153) and anti-C/EBPα (04-1104) antibodies were purchased from Upstate Biotechnologies (Lake Placid, NY, USA). Anti-Osterix (sc-22538), anti-Myc (sc-40), anti-HA (sc-57592), and anti-α-tubulin (sc-53646) antibodies were obtained from Santa Cruz Biotechnology (Santa Cruz, CA, USA). Anti-PPARγ was purchased from Chemicon International Inc. (MAB3872, Temecula, CA, USA). Insulin (I2643), dexamethasone (D4902), 3-isobutyl-1-methylxanthine (I5879), and rosiglitazone (R2408) were purchased from Sigma (St. Louis, MO).

### Transient transfection and luciferase reporter assays

3T3-L1 cells were transfected by polyethyleneimine (PEI; Polysciences, Inc.). The total amount of transfected plasmids in each group was normalized by adding an empty vector. For luciferase reporter assays, cells were transfected with indicated combinations of 0.2 μg of luciferase reporter plasmid (PPRE-Luc or aP2-Luc), 0.1 μg of pCMV-β-gal plasmid, and 0.2 μg of expression vectors (HA-PPARγ, Myc-Osterix, or shOsx). PPRE-Luc contains the consensus PPAR responsive element (PPRE) and aP2-Luc contains the promoter region of the adipocyte fatty acid binding protein (*aP2*) gene that includes PPREs. pCMV-β-gal was co-transfected to normalize transfection efficiency. After 36 h, the cells were lysed and subjected to measure luciferase activities using the Luciferase Reporter Assay Kit (Promega, Madison, WI, USA).

### Oil red O staining of 3T3-L1 cells

On day 8, the extent of adipogenesis was measured by oil red O staining. Briefly, cells were washed three times with phosphate-buffered saline (PBS) and fixed for 30 min with 4% paraformaldehyde in PBS. After three times wash with PBS, fixed cells were incubated with 0.5% oil red O in isopropanol for 30 min using an adequate shaker. Cells were washes with PBS and were photographed under a light microscope for analysis. Oil red O dyes were dissolved in isopropanol and lipid accumulation was quantified by measuring the absorbance at 510 nm using a spectrophotometer.

### mRNA expression analysis

Total RNA was extracted using RNAiso Plus (Total RNA extraction reagent, Takara) and Oligo (dT) primers and reverse transcriptase (Promega) were used to synthesize cDNA. PCR were performed using the synthesized cDNA. The below conditions were applied for PCR: pre-denaturation at 95 °C for 5 min, 25–35 cycles of denaturation at 95 °C for 30 sec, annealing at a specific temperature optimized for each primer pair for 30 sec, extension at 72 °C for 30 sec-1 min, and a final extension at 72 °C for 10 min. Real-time PCR was performed using SYBR Premix Ex Taq kit (TaKaRa Bio, Japan) and a CFX96 real-time PCR System. Samples were incubated at 95 °C for 30 s followed by 40 cycles at 95 °C for 5  and 60 °C for 30 s. Expression level of GAPDH (glyceraldehyde 3-phosphate dehydrogenase) was used as an internal control to normalize mRNA expression. The primers sequences used for PCR are listed in Table 1.

### Immunoblotting and immunoprecipitation

After transfection (36 h), cells were washed with PBS and lysed in a lysis buffer (1% NP-40, 25 mM HEPES (pH 7.5), 10% glycerol, 150 mM NaCl, 25 mM NaF, 0.25% sodium deoxycholate, 1 mM EDTA, 1 mM Na_3_VO_4_, 10 μg/mL aprotinin, 10 μg/mL leupeptin, and 250 μM phenylmethanesulfonyl fluoride). For immunoblotting (IB), Aliquots from the cell lysates containing 30 μg of protein were heated at 95 °C for 5 min and were then separated using SDS-PAGE, and proteins were transferred to PVDF membrane. Blots were blocked for 30 min with Tris-buffered saline (50 mM Tris.HCl, pH 7.4, 150 mM NaCl)-0.05% Tween 20 (TBS-T) supplemented with 5% skim milk. Proteins were visualized using appropriate primary antibodies, horseradish peroxidase-conjugated secondary antibodies, and Immobilon Western Chemiluminescent HRP Substrate (Merck Millipore, Billerica, MA, USA). The visualized membranes were stripped after Western blotting with stripping buffer (0.5 M glycine (pH 2.0) and 10% SDS), followed by re-blocking in TBS-T with 5% milk for 30 min. Detection of α-tubulin was performed as loading control. For immunoprecipitation (IP), centrifuged lysate supernatants were subjected to IP with appropriate antibodies and protein A Sepharose beads. The immunoprecipitated proteins were subjected to SDS–PAGE and visualized by IB.

### Statistical analysis

For quantification of IB and RT-PCR results, band intensities were measured using Multi Gauge, V3.0 image analysis software (FUJIFILM, Tokyo, Japan). The results were expressed as the means ± SD. We determined the statistical significance of the difference between experimental groups in instances of single comparisons by the two-tailed unpaired Student’s t-test of the means. For multiple means comparisons, one-way analysis of variance (ANOVA) with the Bonferroni post hoc test using Sigma plot.

## Additional Information

**How to cite this article**: Han, Y. *et al*. Osterix represses adipogenesis by negatively regulating PPARγ transcriptional activity. *Sci. Rep.* **6**, 35655; doi: 10.1038/srep35655 (2016).

## Figures and Tables

**Table 1:** Sequences of primers used for PCR.

	Gene	Primer sequence (5′→3′)
PCR	*Osterix*	(F) 5′-GGC ACA AAG AAG CCG TAC TC-3′
		(R) 5′-GCC TTG TAC CAG GAG CCA TA-3′
	*C/EBPβ*	(F) 5′-AGC CCC TAC CTG GAG CCG CT-3′
		(R) 5′-GCG CAG GGC GAA CGG GAA AC-3′
	*C/EBPα*	(F) 5′-TGC TGG AGT TGA CCA GTG AC-3′
		(R) 5′-AAA CCA TCC TCT GGG TCT CC-3′
	*PPARγ*	(F) 5′-ATC AGC TCT GTG GAC CTC TC-3′
		(R) 5′-ACC TGA TGG CAT TGT GAG AC-3′
	*GAPDH*	(F) 5′-ACC ACA GTC CAT GCC ATC AC-3′
		(R) 5′-TCC ACC ACC CTG TTG CTG TA-3′
qPCR	*Osterix*	(F) 5′-AGC GAC CAC TTG AGC AAA CAT-3′
		(R) 5′-GCG GCT GAT TGG CTT CTT CT-3′
	*PPARγ*	(F) 5′-GCC CTT TGG TGA CTT TAT GGA-3′
		(R) 5′-GCA GCA GGT TGT CTT GGA TG-3′
	*aP2*	(F) 5′-GAA CCT GGA AGC TTG TC-3′
		(R) 5′-CGT GAC TTC CAC AAG AGT-3′
	*Adiponectin*	(F) 5′-CCC AGG ACA TCC TGG CCA CAA-3′
		(R) 5′-CCT TCA GCT CCT GTC ATT CCA-3′
	*GluT4*	(F) 5′-GTA ACT TCA TTG TCG GCA TGG-3′
		(R) 5′-AGC TGA GAT CTG GTC AAA CG-3′
	*GluT1*	(F) 5′-TCA ACA CGG CCT TCA CTG-3′
		(R) 5′-CAC GAT GCT CAG ATA GGA CAT C-3′
	*GAPDH*	(F) 5′-AGG TCG GTG TGA ACG GAT TTG-3′
		(R) 5′-GGG GTC GTT GAT GGC AAC A-3′
